# Expanding the Conceptualization of Support in Low-Wage Carework: The Case of Home Care Aides and Client Death

**DOI:** 10.3390/ijerph19010367

**Published:** 2021-12-30

**Authors:** Emma K. Tsui, Marita LaMonica, Maryam Hyder, Paul Landsbergis, Jennifer Zelnick, Sherry Baron

**Affiliations:** 1 Graduate School of Public Health & Health Policy, City University of New York (CUNY), New York, NY 10027, USA; Marita.LaMonica@sph.cuny.edu; 2 Barnard College, Columbia University, New York, NY 10027, USA; mah2335@barnard.edu; 3 Department of Environmental and Occupational Health Sciences, School of Public Health, State University of New York (SUNY)-Downstate Health Sciences University, New York, NY 11203, USA; Paul.Landsbergis@downstate.edu; 4 Graduate School of Social Work, Touro College, New York, NY 10001, USA; jennifer.zelnick@touro.edu; 5 Barry Commoner Center for Health and the Environment, Queens College, City University of New York (CUNY), New York, NY 11365, USA; Sherry.Baron@qc.cuny.edu

**Keywords:** occupational stress, social support, home care aides, carework, social ecological framework

## Abstract

Home care aides are a rapidly growing, non-standard workforce who face numerous health risks and stressors on the job. While research shows that aides receive limited support from their agency employers, few studies have explored the wider range of support that aides use when navigating work stress and considered the implications of these arrangements. To investigate this question, we conducted 47 in-depth interviews with 29 home care aides in New York City, focused specifically on aides’ use of support after client death. Theories of work stress, the social ecological framework, and feminist theories of care informed our research. Our analysis demonstrates aides’ extensive reliance on personal sources of support and explores the challenges this can create in their lives and work, and, potentially, for their communities. We also document aides’ efforts to cultivate support stemming from their home-based work environments. Home care aides’ work stress thus emerges as both an occupational health and a community health issue. While employers should carry responsibility for preventing and mitigating work stress, moving toward health equity for marginalized careworkers requires investing in policy-level and community-level supports to bolster employer efforts, particularly as the home care industry becomes increasingly fragmented and non-standard.

## 1. Introduction

Non-standard work—including temporary, agency-based, contract, and gig work—is increasing [1], with urgent implications for how we conceptualize worker support. From the perspective of societal health, one important example of non-standard work is provided by home care aides whose labor takes place outside of typical worksites and working hours, further distancing it from “traditional” work arrangements.

Home care aides, including personal care aides, home health aides, and home-based nursing assistants, comprise one of the fastest growing job sectors in the United States due to the increasing older adult population and ongoing deinstitutionalization of care [2]. They provide services that help older adults and disabled individuals to remain in their homes. They support clients’ activities of daily living, such as eating, toileting, bathing, and moving around, and also engage in meaningful and complex relational work with clients and their families [3,4]. Aides are among a larger cadre of careworkers supporting individual and societal health. Broadly defined, careworkers do “various kinds of work—mental, manual, and emotional—aimed at providing the historically and socially, as well as biologically, defined care necessary to maintain existing life and to reproduce the next generation” [5].

Multiple dynamic factors create precarity, job insecurity, stress, and poor health outcomes for the home care aide workforce. First, the structure of the home care industry is highly fragmented, leading to challenges for organizing and advocacy. At the same time, the industry is largely federally funded through Medicaid, which has kept wages low, with a median wage of $11.52 per hour in 2019 [2]. In the United States, the home health aide workforce is primarily female, and disproportionately comprised of women of color and immigrants. Specifically, aides in the U.S. are 87% female, 28% Black, 23% Hispanic/Latinx, and 8% Asian or Pacific Islander. They are also only 69% U.S. citizens by birth [2]. Many experience significant poverty. Nearly 20% of aides live in a household that falls below the federal poverty line, and almost half of aides live in low-income households earning 200% of the federal poverty level or less [2]. In part, low earnings are due to unpredictable schedule changes and the part-time hours that many aides are assigned. This work is also widely acknowledged as involving multiple forms of stress, including both physical strain and hazards and emotional strain from navigating client relationships and clients’ home social environment [6,7,8]. As a result, the general, physical, and mental health of home care aides as a population is poorer than that of similar low-wage worker populations [9].

While home care aide stress has been well-documented, in this article, we explore the forms of support aides use to buffer and navigate work stress. Since the 1990s, support at work has been considered a key lever in workers’ stress and well-being on the job. Notably, support in work stress models centers on support provided by employers, as an acknowledgement that employers should take responsibility for supporting employees to reduce work stress. Karasek first conceptualized the importance of workplace support as a critical addition to his conceptual job strain model (now called the Job-Demands-Control-Support model or JDCS), which showed that jobs with high demands result in less job strain in the presence of control and support [10]. However, some have critiqued the utility of the job strain model for home care aides, noting that intrinsic rewards and pride in the work are not adequately accounted for [8]. The Effort–Reward Imbalance model might be more appropriate for conceptualizing the rewards of caring labor in relation to job stress. This model details rewards of work spanning income, job security, respect from supervisors, and opportunities for advancement [11]. With support considered a reward of work though, this model is limited in its ability to conceptualize the effects of different kinds of support, or their interaction with efforts. In addition, “support” has not been included in more recent shorter versions of the Effort–Reward questionnaire [12]. The JDCS model also includes coworker support. However, since home care labor takes place in an atypical workplace, the interaction and blending of a variety of forms of support—not just standard job-based supports—requires careful attention if we are to fully understand the resources that aides draw on to sustain themselves.

In order to explore these topics, we examine the experiences of home care aides in New York City with a particular stressor, client death. Client death is a useful focal point because it can lead to both substantial emotional stress from the loss of a close relationship and job insecurity while awaiting a new client, which can also interact with and amplify each other [13]. As a result, client death experiences often require aides to seek out multiple forms of support from a variety of sources. While much of the literature on home care aides acknowledges client death as an important, and often problematic, feature of work for aides [3,8,14], home care employers have generally done little to support aides explicitly around these events, which are a regular part of the work [15]. A mixed-methods study examining how aides perceive support from agency staff and coworkers around client death showed that a minority of aides reported that these forms of support were available to them, but that almost all who utilized support found it to be effective in addressing their stress related to client death [16]. It is not surprising that workplace support related to client death is lacking, given that minimal job-based support for home care aides generally is a pervasive issue [17,18,19]. As a result, aides must supplement job-based forms of support with other resources. Our article asks: How do aides make use of a variety of sources of support to navigate client death, and what problems arise as a result?

### Theoretical Orientations

We ask these questions with the goal of better understanding how support that prevents or mitigates job stress works in practice. Importantly, we also seek to bring the complexities of support more vividly into view through the models we use to guide our research. Toward this latter goal, our research is informed by three bodies of theory. The first of these are the work stress models, such as the JDCS and the ERI, discussed above. These theories provide the primary frame for our interest in expanding the notion of support to include support beyond the job in order to better reflect the realities of carework and of non-standard low-wage work. Notably, the only non-job-based support that has been included in work stress models thus far is what has been called “personal resources” [20]. However, these are characteristics at the individual level, such as self-efficacy, and the models currently do not recognize the social systems that strongly shape individual-level personal resources. To move to the level of social systems, we are relying on a second theory, the social ecological framework, widely used in public health research and practice, which articulates the multiple levels and social systems that shape health and their interactions [21]. Finally, this work is informed by feminist carework scholars who draw our attention to the idea that anemic systems of job-based support for home care workers, and other low-wage careworkers, have a powerful historical context [22,23]. Specifically, we recognize that home care labor and other forms of carework have only recently been acknowledged by the federal government as constituting work that merits protection under the Fair Labor Standards Act. The exemption of home care labor from such protections and supports for so long is the direct result of racist economic systems and policies that continued in the wake of emancipation, and the legacy of racialized ideas about who provides care, why, and how still thrives in how we conceptualize support for careworkers [24]. In addition, the concentration of women in these forms of labor expresses the history of women’s oppression in society, within and outside of the labor force, and the “cultural construction of care as an especially important moral obligation for women” [25]. Thus, we understand who provides home care labor and how they are supported as functions of and inextricable from the intersections of race, class, and gender oppression [26].

## 2. Materials and Methods

### 2.1. Design

In this study, we conducted an inductive, theory-informed thematic analysis in keeping with the realist approach described by Ritchie and colleagues [27]. Specifically, we used in-depth interviews with home care aides to explore their experiences with support after client death. The study began in October 2019 and thus was conducted prior to and during the COVID-19 pandemic. We ceased data collection February–May 2020 in the early months of the COVID-19 pandemic in New York City. We then conducted remote data collection from June–September 2020 when COVID-19 rates were lower in New York. Ultimately, we conducted 47 in-depth interviews with 29 English-speaking home care aides.

### 2.2. Sampling and Recruitment

We purposively sampled participants who had worked for at least one year as an aide and had experienced at least one client death. Because of resource limitations, we only sampled English-speaking aides though we sought to sample aides who were born in the US and outside of the US, as well as aides who were Black and Latinx (See Table 1). We conducted recruitment in person during in-service training sessions at two home care agencies in New York City from October 2019 to January 2020 (*n* = 27). After that, we continued recruitment via snowball sampling with a particular focus on purposive sampling of additional Latinx aides (*n* = 2). In Summer 2020, we also recontacted aides who had participated in October 2019–January 2020 to better understand aides’ experiences with client death, stress, and support during the pandemic (*n* = 18).

### 2.3. Sample Description

A description of the resulting sample can be found in Table 1. Participating aides were female, majority Black (65%), with mixed race individuals, white individuals, and Asian individuals making up the remainder of the sample. Twenty percent of aides (*n* = 6) identified as Latina. Aides had a wide range of experience in home care, from 1 to 27 years, and were a mix of those with limited or no experience working on hospice cases, and those with more hospice experience. Almost all aides identified as religious (*n* = 27), and a majority of these identified as Christians.

### 2.4. Data Collection

Initial interviews were in-person and mostly conducted on site at agencies, while Summer 2020 interviews took place via phone or Zoom due to pandemic restrictions on in-person data collection. All interviews were semi-structured with substantial opportunity for participants to suggest the direction of the interview. A guide helped structure these interviews (see Supplemental File S1) and evolved with time. For the Summer 2020 interviews, for instance, we added questions about experiences and support during the pandemic.

### 2.5. Analysis

Two authors (ET and ML) led the development and refinement of coding schemes for the two sets of interviews (initial and summer), which were based on close readings of the transcripts and fieldnotes. Transcripts were then coded in Dedoose, and coding schemes continued to evolve through regular consultation among coders. The analysis presented here focuses on the sources and types of support that aides used. The importance of problems that arose in using support emerged during analytic memo-writing and team interpretation of coded data. Finally, we used a range of strategies to increase quality including practices of reflexivity (on the part of participating coders), triangulation (via multiple analysts and multiple interviews), and peer debriefing with experienced authors outside of the coding team (SB, PL, and JZ) [28]. All study procedures underwent review by the CUNY Graduate School of Public Health and Health Policy’s IRB.

## 3. Results

In the sections that follow, we describe in detail the sources of support that aides used to navigate client death. We explore job-based support, personal support, and sources that blend the professional and the personal. We examine the key features of each support category and aides’ experiences with these sources of support. We then focus on two of the problems inherent in relying so heavily on personal and blended source of support. Finally, we briefly describe the changes in support we documented during the COVID-19 pandemic. Note that, because aides may develop and refine their support-seeking over time, we show each aide’s years of work experience when presenting data.

### 3.1. Job-Based Sources of Support

Of the various types of support available to aides in relation to client death, the literature has focused most closely on the job-based sources of support, as described earlier. Like this literature, our participants emphasized the important role that coordinators, the agency staff who assign and manage cases, can sometimes play in providing both emotional support and instrumental support [29] that provides behavioral or material assistance in addressing problems. We also considered training, agency programs and policies, and union-based sources of support mentioned by aides to be job-based supports. Because this section largely replicates what is already known about job-based support for client death [13,16], we present these data in Table 2.

### 3.2. Personal Sources of Support

Far more commonly used by aides in our study were personal sources of support. We define personal sources of support as support that is fully outside of the domain of work—for instance, from the aide’s family, friends, or religious community. When these supports were present, particularly family support, aides’ experiences with client death overall seemed far less negative. In keeping with definitions of personal resources in existing work stress models, we also include ways that individuals supported and relied on themselves in this section.

#### 3.2.1. Family and Friends 

Aides’ family members and friends were sometimes the sole source of support for aides. Aides noted multiple, general ways that these individuals help them cope emotionally. As one aide said, “My immediate family [is a source of support after client death]. I go home and I talk to my husband and be with my kids, you know, it just definitely helps me to deal with it” (Participant 210, 8 years). Another emphasized the singularity of this source of support: “You know, friends and family are always there for support. But basically, that’s it” (Participant 221, 3 years). In particular, some aides mentioned other care workers in their families and social networks whose similar experiences gave them a unique lens through which to offer support. As one aide said, “I do have an aunt who’s a nurse and she’s experienced death on many occasions, so that’s very helpful.” (Participant 205, 5 years).

In some situations, support from family and friends seemed sufficient after a client’s death. In other cases, family members could dismiss an aide’s feelings or otherwise not adequately attend to them. One aide suggested the inadequacy of her family’s response: “They only say I’m sorry to hear about your passing of a patient.” (Participant 209, 10 years). Another shared the experience of family members with a more directly antagonistic view toward the work: “Support with me wasn’t in my family cuz they don’t care. My son [says], all of you need to quit. I said, ‘Well, [are] you gonna pay my rent?’” (Participant 102, 9 years).

#### 3.2.2. Religious Support

Religious practices—prayer, most commonly—and religious communities were also discussed as central forms of support for many aides following client death. While aides spoke frequently about praying on their own, others sometimes looked for company through prayer, as in the case of this aide: “The spiritual approach is that there is always help available. Personally, there’s a prayer hotline that can be used when I can’t go within myself to do it [e.g., cope with client death]” (Participant 217, 2 years). When possible, attending church further underscored the availability of help: “Yeah, it took me about a week [to recover from a client’s death], because I had to go to church. I go to church and it rejuvenates me. If I can talk to the pastor, then that helps me, because they always help me to take myself out of the equation” (Participant 213, 7 years). Another aide described a difficult experience with a client whose case had included Sunday hours and had thus prevented her from attending church for some time. In going back to church, she said, “That’s where I started to feel much relief” (Participant 201, 10 years).

#### 3.2.3. Self-Support

Aides also spoke often of using individual coping approaches and beliefs to support themselves through client death. In addition to prayer, as discussed above, there were two primary mechanisms of self-support in our data: Keeping busy and making peace with client death. Several aides boisterously listed the many activities in which they involve themselves to help keep busy and move on from a client’s death. One aide said that she can “make [her] own therapy” in this way. “You read a book, you do some game, you watch TV, you put the radio, you know, you dance. […] I always have something to do. And then if I don’t have something to do, I look for, you know, like something—let’s go outside” (Participant 105, 13 years). Others found ways to address the death more directly and come to terms with it. As one aide said, “I can get myself together very fast, by considering that, you know, this is their time and they have to go” (Participant 219, 14 years). Another spoke about how suffering at the end of life helped her accept death: “If people are dying, and they’re really, really suffering, just let them go” (Participant 211, 4 years).

#### 3.2.4. Personal Support for Financial Impacts of Client Death

Personal sources of support, and especially reliance on oneself, were also the only forms of support discussed when it came to the financial implications of client death. Though aides sometimes described loss of income after these events, they largely accepted it as part of the job. As one aide said, “Did I see a change [after the client’s death]? Let me see. Just losing another client. Losing a client the money went down a little bit, you know what I’m saying?” (Participant 211, 4 years).

The primary tactic that aides described for navigating the financial implications of client death was to actively pursue new cases at the agency if they are not quickly reassigned. If aides were not successful, then they might seek work opportunities at another agency, and many aides in our sample worked at least some of the time at other agencies in order to secure sufficient hours. A few also described engaging in other forms of work as well: “Well, if I needed financial support, I made—I had to find a way to get it. So, with the other skills that I have I would create additional income. I do counseling and nutrition counseling, workshops, lectures, things like that, so I was able to take care of my financial needs.” (Participant 221, 3 years). For some aides, family members provided a sense of financial security “my husband has a very good job” (Participant 209, 9 years).

Aides might want to take time off after client loss but are typically unable to do so, due to financial pressures. As one said, “I’m not able to take off any time, because of the money that I earn is not enough to have, to even do anything beyond basically basic needs” (Participant 217, 2 years). When aides do take time off, they often see it as their choice to go unpaid, despite the need for time off stemming from the work. As another described, “No, I didn’t get support from [the agency]. […] I just took my days off. I chose to lose the pay, right. Because I just—because death is real and it’s fragile. Right? And you need a moment to hit the reset” (Participant 215, 3 years).

### 3.3. Blended Sources of Support

For most home care aides, the workplace is the client’s home. While some support stems from this work environment, we do not consider this to be “job-based support” for aides, as it is not employer- or union-generated. Rather, we use the term “blended” to acknowledge that while relationships developed in the home with clients, their family and friends, and sometimes with co-workers do emerge from a professional context, they often require active cultivation and interaction outside of the workplace or work hours to function as sources of support. Hence, aides are blending job-based and personal approaches to support. This is a phenomenon that is unique to some types of non-standard work, as coworker and client relationships are more squarely within the context of job-based structures for standard workers.

#### 3.3.1. Co-Workers

While aides only rarely work on-site together at the same time, aides working for the same agency can sometimes serve as sources of support. Aides described finding support among their peers at in-service classes and sometimes sharing client loss experiences in those settings. They occasionally built friendships that continued outside of trainings, staying in touch through texting, calling, Whatsapp, and, sometimes, in-person gatherings. They described these relationships as limited but usually positive. As one aide who stayed in touch with coworkers by phone and occasionally went out for lunch with them said, “My coworkers actually, you know, they always reach out to each other and give each other support” (Participant 210, 8 years). We also saw glimmers of the vibrancy of some coworker interactions while we conducted recruitment during in-service trainings: Aides sharing their experiences during class, swapping stories over lunch, saving a seat for someone.

However, some aides gestured at the ephemerality and fragility of these relationships in their interviews. As one experienced aide said, “I had this one girl [coworker who she stayed in touch with], but I don’t talk to her no more. Like—cuz I don’t like to talk to people and they turn the conversation on themselves when I’m generally trying to get something out [of the conversation]” (Participant 206, 20 years). Another aide mentioned developing a group of aide friends during her initial training. The aide said that the group stayed in touch for a while but eventually they grew apart “because we all have our different problems and cases. You don’t have enough time. Families and so on” (Participant 203, 3 years).

#### 3.3.2. Deceased Client’s Family and Friends

Aides sometimes spoke about meaningful experiences of reciprocal emotional support between themselves and family members or friends of the deceased client. Our data indicate that aides might contact a client’s family after client death (or vice versa) and find support through shared grief, revisiting memories, and ongoing social interactions. Sometimes this was through a one-on-one interaction, as in this aide’s experience: “I felt really sad. I don’t know if they [the agency staff] know, but I immediately called the cousin and said I’m so sorry about what happened and that she was a really good person. […] And her cousin was like, “Oh, she spoke so highly about you. She loved you” (Participant 101, 1 year). Other aides talked about more collective experiences, as in this case: “The family had a little get together and they invited us [the aides], so we were there sharing her spirit and talking about her. So that was a thing in terms of coping, that little get together” (Participant 216, 8 years).

In many cases, this support was short-term as “everybody moves on with their lives” (Participant 103, 17 years), though we also heard about aides who were in touch with the deceased client’s families in a more ongoing way. For instance, one aide developed a particularly close relationship with her deceased client’s friends. She said, “…you can talk and we laugh about the things that she [the client] used to say. I think if I hadn’t been in touch with them, it would’ve felt sad and lost. […] I would’ve taken it on more” (Participant 202, 20 years). It had been a difficult case over three years, with multiple hospital stays interspersed among periods of home care. Throughout this time, this aide had worked together with the client’s neighbors and ex-husband to provide care and stay in touch. After the client’s death, they checked in on each other, spent time together, and cooked for one another. Our interview was nine months after the client passed away and this aide reported that she was still in touch with members of this group at least weekly.

Note that, in general, aides who had been in the profession longer (and who had thus managed to stay in the profession) appeared to draw from a wider variety of techniques for supporting themselves (which we call “self-support” above) and moving on from client death. Other sources of support and the ways these were navigated did not vary markedly by aides’ amount of time in the profession.

### 3.4. Problems with Relying on Personal and Blended Sources of Support

In addition to demonstrating the importance of personal sources of support and, to a lesser degree, blended sources of support, our data suggest two core problems that might arise in a system that relies on these so centrally: (1) The potential for these venues of support to increase job stress and threaten job security for aides, and (2) the absence of personal and blended support that some aides face.

Because these forms of support require operating in a space between professional and personal, they had the potential to create problems with aides’ employers who want the lines between professional and personal to remain clear and impermeable. Our data provide one overt case in which using these forms of support threatened an aide’s job security, but we heard many subtler examples of the stress aides carry in navigating this personal–professional divide. One aide, for example, grieved a client deeply and despite support from friends and family, the persistent pain from this loss led the aide to post a picture of herself with the client on Facebook, without a name and with the words “Rest in peace”. As the aide said though, “I was just so distraught and I was looking for relief, and I’m not going to lie to you, for some reason, that gave me relief” (Participant 218, 16 years). This was a highly contested practice within the agency context, however. The aide described how another aide reported this post, and as a result, she was suspended from work for three days while the agency investigated the situation. She feared being fired, though ultimately, she was not.

While this situation was likely problematic from an agency perspective due to concerns about patient privacy, agency staff strongly discourage relationships between aides and clients and their social circles generally, as has been documented in the literature [3,13,15]. As one aide in our study put it, “We are so many times told, don’t be so close [with clients and their families]” (Participant 103, 17 years). We can thus hear the more subtle stressful experiences of navigating this divide in the quote from participant 101 above. She said, “*I don’t know if they [the agency staff] know*, but I immediately called the cousin…” (italics added). Her attentiveness to whether agency staff were aware that she had broken the rule about contacting family members after the case ended suggests that this might have weighed on her mind. However, if this aide had tried to follow agency prohibitions, she would not have been able to use this meaningful source of support. The same goes for the others in our sample who used relationships with clients’ families and friends to navigate and soften the experience of client death.

Some aides might not have extensive personal support networks, might not feel comfortable using them, or might lack the ability, willingness, wherewithal, or comfort (given agency warnings against these relationships) to cultivate blended sources of support. In the absence of job-based support, and lacking these additional forms of support, aides might be left to navigate a difficult experience alone. One aide illustrated vividly how useful support outside her personal network would be in these situations: “It would have been a good help, you know, if there was some kind of—somebody I could—that’s not my family or, somebody I could bounce things back off again, that’s more support. Somebody outside of my scope of friends and family. Somebody neutral that, you know, that doesn’t know me, that I could just open up to it. And speak about it, and cry or grieve, or if I had to.” (Participant 221, 3 years)

**Figure 1:**
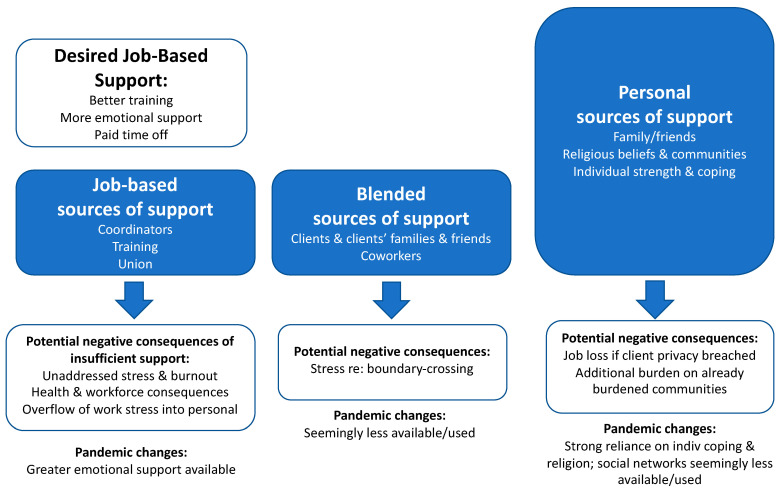
Sources of support used by home care aides to manage work stress from client death.

We summarize our findings on the sources of support discussed above in Figure 1, noting the levels of use of each category of support (denoted by the relative size of each support box), potential negative consequences of using these forms of support, some of the job-based forms of support that aides spoke of desiring, and a brief summary of the shifts we saw in each category of support during the summer pandemic months.

### 3.5. Changes to Aide Support Experiences during the Pandemic

During our Summer 2020 follow-up interviews with aides, we asked about the forms of support aides were using while working during the pandemic. Their responses indicate that non-job-based forms of support remained prominent, religion in particular, and that new, though limited job-based forms of support had arisen in the form of phone-based meetings with other aides organized by their agencies and unions. Support from coworkers and clients and/or their families were barely mentioned in these interviews, suggesting that these forms of support might be particularly likely to vanish under pressure given the effort they take to cultivate. Finally, we note that emotional and financial support from friends and family remained central but appeared more strained in these data. One quote from an aide who lost a client to COVID-19 expresses this vividly: “Everybody is busy, everybody is thinking about the pandemic, everybody has different problems and I don’t want to call any friends and say, ‘Oh, my patient died.’ You know, nobody listen. Yeah, nobody say, ‘Oh, I’m sorry.’” (Participant 003, 2 years) We also heard from aides whose spouses, often the primary breadwinners, were out of work.

Reliance on one’s own strength and coping, and on a relationship with God, were strong narratives in these data. As one aide said, “I don’t need much, because I’m pretty self-motivating and pretty much I don’t get saddened too much. I have my own system that I use to help keep me up […] but I think something like that, a better relationship with your [coordinator] or the nurse, would be helpful” (Participant 221, 3 years). For many aides, faith in God’s ability to protect them offered some relief from the anxiety of continuing work during the pandemic. Conveying the sentiments of many aides who we interviewed, one aide said, “For me, my anchor really is in Christ, the Father, Son, and Holy Spirit. They really help me, because if I get too out of balance or I keep something on my mind too long they just calm my concerns” (Participant 215, 3 years).

A new job-based source of support mentioned by many aides was agency- and/or union-sponsored phone-based support groups and town hall meetings. These sometimes involved hundreds of aides. Some aides noted that these were impractical while they were with clients, too lengthy, not a venue in which they were comfortable, or simply not of interest. Others were more enthusiastic, feeling that the calls offered recognition of the importance of their work, addressed their problems to varying degrees, and offered a chance to vent and a place to better understand the difficulties that other aides were facing. One particularly optimistic aide saw these calls as signs of possible increased support from agencies going forward, saying, “This is the first period—since COVID—that we’ve had a chance to really be in touch with anybody [at the agency] on a personal level, to let them know what we’re feeling, what we’re going through. So, a lot of these things I think they’re working on fixing so that, you know, we could have more support. […] With the support calls, they’re taking ideas or suggestions that we give on those calls, to see how far they can go with it and to create some kind of change in the organization” (Participant 221, 3 years). Other aides appeared less optimistic about the durability of these new forms of support.

## 4. Discussion

Focusing on a single work-based stressor that has both emotional and financial dimensions (i.e., client death), we have shown that home care aides draw heavily on their own internal resources and personal social networks in order to address work stress. They also cultivate and maintain sources of support that cross professional-personal boundaries, which can be valuable but require effort and can create job insecurity and their own forms of stress. In contrast, aides in this study derived only minimal and sporadic support from their employer agencies. How aides navigate in the absence of reliable employer support is especially important to attend to as the home care industry in the U.S. moves towards models in which traditional employer agencies are completely absent or less present. For instance, recently, there has been significant growth in consumer-directed models of home care, in which family members or friends serve as paid home care aides, as well as growth in technology-enabled home care services that facilitate the direct hiring of aides by clients and their families [30].

Our research thus highlights the importance of understanding workers and work stress in the context of their lives and communities, not simply their jobs, and provokes us to consider more deeply how work stress models can be integrated with the social ecological framework for non-standard workers [31]. Specifically, our findings lead us to suggest (1) a rethinking of existing work stress models that better reflects home care aides’ unique working conditions and sources of support, and that integrates the social ecological framework, (2) a new concept we are calling “social accommodation” to theorize the effects of limited job-based support at the population level, and (3) a range of policy- and employer-level recommendations aimed at reducing aides’ work stress through increased support.

### 4.1. Work Stress Models

**Figure 2:**
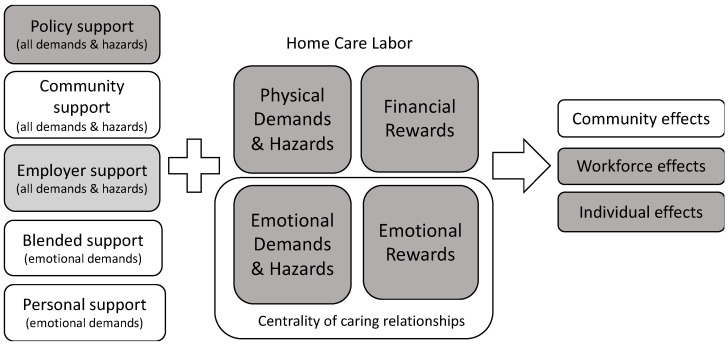
Types of support for carework labor experience and levels of effects. Note: Gray boxes are phenomena documented in the literature. White boxes are model elements that this study adds.

Our findings indicate that home care aides receive limited emotional and financial job-based support, and that more supportive infrastructure on the job is essential to reducing their work stress. However, our research also suggests the urgency of recognizing that some support addressing work stress will continue to come from outside of work, due to the general movement toward less standard work arrangements. Thus, we propose that existing models of work stress should better incorporate the wide range of supports workers actually draw on in managing work stress, supports that could be bolstered. In Figure 2, we propose a new model of home care aide work stress that emphasizes the broader sources of support used by home care aides, the types of support they need, and the effects of current patterns of support.

In this model, we conceptualize the key demands and rewards of home care labor as being physical, emotional, and financial. Similar factors were recently described in a qualitative meta-synthesis of home health aides’ occupational health experiences that included studies from the United States, Canada, Norway, Denmark, Ireland, and Sweden [32].The emotional demands and rewards are particular to carework, given the centrality of caring relationships on the job, and are pronounced among aides who work in the home without regular coworker support [3,4]. Alongside essential job-based supports, our model first integrates the kinds of personal and blended sources of support described in this article. These sources of support might be especially useful for navigating emotional demands. We also include community and policy supports that could address multiple demands and hazards of home care work. We envision community supports as programs and practices that allow community-based organizations (such as churches and worker organizations) and individuals to better respond to and buffer the emotional dimensions of work stress (i.e., emotional demands and hazards), while also creating spaces for activism, including around the non-emotional dimensions of work stress (i.e., physical hazards and financial rewards). Policy supports serve to strengthen the social fabric that sustains workers and their communities and can also buffer work stress. All support levels in the model have the potential to work in concert, as in the social ecological framework. For instance, in the case of client death, if aides had access to more paid leave (policy support), employers could then facilitate the use of leave as well as provide better recognition of the loss, and grief support, if needed (employer support). Strengthened community-based organizations, worker organizations, and unions could assist aides in dealing with the emotional experience of client death during this leave time, help connect them to peers, and ensure they know their rights (community support).

Another contribution of this model is to highlight the complexities and contributions of blended sources of support for home care aides, not currently recognized in work stress models. Employers have historically done little to actively bolster coworker support, and existing supervisory relationships often increase aides’ work stress [3,13]. In the absence of such support, aides seek other social support at the worksite via clients, their family members, and friends. These relationships can be their own rich resource, allowing workers to derive dignity and interpersonal recognition for difficult, low paid work. However, these relationships might also pose risks to workers, in which workers “overexert” themselves emotionally, physically, and/or economically because of their commitment to clients [8,18,33]. These dynamics are particularly important to attend to as consumer-directed models of home care grow. Recognizing this, home care agencies should develop new training and support systems that directly face these complexities and honor the needs of both aides and clients [34]. Currently, agencies appear to treat these relationships as merely conferring risk and they advise aides to avoid developing close relationships with clients [13,15], in spite of the fact that rapport and trust are critical to aides’ ability to provide quality care [18].

### 4.2. Social Accommodation

Drawing on the social ecological framework, we also note issues at the population level that might arise from the heavy emphasis on personal support, rather than job-based support, to buffer job stress (see “community effects” in Figure 2). The home care aide workforce is disproportionately comprised of women of color and immigrant women [2], and as a result, aides are often members of marginalized communities where resources and social support might already be strained. One striking example of this has been documented by Sociologist Debra Umberson who has shown that black Americans are at greater risk of losing immediate family members earlier in the life course than white Americans. Umberson argues that these death exposures diminish social resources, and thus that “Black deaths matter” to understanding health inequities [35]. This is even more the case now, in light of the dramatically inequitable impact of the COVID-19 pandemic on communities of color [36], and particularly women of color who do the majority of frontline carework. Both our research here and other recent research shows that aides have been using their personal resources and social networks to navigate the challenges of the pandemic period [37]. While our data in this study cannot speak to this directly, we wonder, how does this additional overflow of work stress impact communities that are already stretched, saddled with loss, and challenged on so many fronts? How does it impact women who are so often sought out for emotional support, in particular? Perhaps also, how have these challenges shaped and perhaps strengthened social networks in these communities? Thus, our research begins to point to the possible higher-level population effects of minimal job-based support on aides’ communities. Anthropologist Elana Buch identified the many forms of “bodily accommodation” that aides engage in to deliver good care [38]. We wonder if it might be appropriate to call the reliance on social support beyond the job for navigating work stress among aides a kind of “social accommodation” that aides’ families, friends, and communities (and sometimes client’s families) are making. If we find that this social accommodation is costly, we then have further evidence of the urgency of providing more support to workers, whether these supports are demanded or not, to promote and protect societal health equitably.

### 4.3. Policy and Employer Recommendations

Our findings suggest a need for both worker-focused and community-focused policy action, as well as steps that employers should take. Worker-focused policies that could help to address aides’ lack of support and resultant health inequities include paid leave, living wage, affordable health care, and strong worker health and safety policies [39]. Notably, the Build Back Better Act currently under debate in the Senate may substantially expand funding for home care while improving workers’ wages and working hours [40], which would offer both agencies and aides much needed support. A range of additional policies are needed to bolster community support systems, however, and to respond to the potential for social accommodation described here. These include antiracist, feminist, and inclusive labor, education, housing, food, and immigration policies, among others.

For employers, our findings indicate that more and better job-based support is needed. Importantly, such support has been shown to be effective when present [16], and it is worth noting that countries such as Holland have experimented with multiple forms of increased job-based support within home care organizations broadly [41]. Our study also suggests how important it is for job-based support to take place in the context of more realistic, transparent, and supportive understandings of personal–professional boundaries on the part of agencies. As sociologist Clare Stacey has argued, greater support for aides’ emotional labor should be developed, rather than efforts to restrict such labor [4]. In addition to training for aides on navigating boundaries with clients and their families and training for coordinators on providing empathic support for aides, we recommend the development of job-based emotional support systems for aides that are separate from case assignment processes, as we saw emerging during the pandemic in this study. Such systems could offer spaces for listening and support that would not have repercussions for assigned hours and job security. Notably though, the current state of home care financing and the industry’s fragmentation undermine the ability of many agencies to cultivate such support among their staff [42]. Enhancing the ability of co-workers to help one another to navigate and cope with the emotional demands of work, perhaps through computer-mediated modes, may also be a promising approach [43].

Finally, this study has some limitations. Most significantly, our sample is not representative of the diverse experiences of aides throughout the U.S. First, we included only aides who marshalled enough support to be able to continue in the job after one or more client deaths. We are thus not able to speak to the support-related experiences of aides who left the workforce, or to assess differences in support. Second, aides were primarily recruited from two agencies in New York City at which aides are unionized and thus likely had more employer/union support than aides have generally. Finally, aides were English-speaking and thus our data do not represent the perspectives of aides who are non-English-speaking and who might have different support systems outside of work. In considering transferability, while our findings are centered on home care aides, we expect that some of these ideas might have relevance for the larger population of low-wage careworkers who also typically lack job-based support. Finally, given our model’s focus on the emotional health and well-being of people whose jobs routinely put them in contact with grief and loss, our research might also be relevant to a range of carework professions, particularly in the wake of the COVID-19 pandemic.

## 5. Conclusions

Home care workers are a rapidly growing workforce whose importance has only increased as a result of the COVID-19 pandemic. In order to build and support this workforce in ways that allow our society as a whole to thrive, we need to better understand the kinds of support that workers draw on in order to provide this critical form of labor, as well as the effects of working with current patterns of support. Our analysis indicates aides’ extensive reliance on personal sources of support, and on some blended personal-professional sources of support, which can create challenges in their lives and work, and, potentially, for their communities. Job-based support currently plays a limited and unreliable role in helping aides to navigate stress from client death. Home care aides’ work stress thus emerges as both an occupational health and a community health issue. While employers should carry responsibility for preventing and mitigating work stress, moving toward health equity for marginalized careworkers requires investing in policy-level and community-level supports to bolster employer efforts, particularly as the home care industry becomes increasingly fragmented and non-standard.

## Figures and Tables

**Table 1:** Sample description (*n* = 29).

Age		
	Median	53
	Range	30–67
Gender		
	Female	29 (100%)
Race		
	Black	19 (65%)
	Mixed	4 (14%)
	White	4 (14%)
	Asian	2 (7%)
Ethnicity		
	Hispanic	6 (20%)
Country of birth		
	Foreign-born	17 (59%)
Home care experience (years)		
	Median	8
	Range	1–27
Hospice experience		
	None	6 (21%)
	Minimal	10 (34%)
	Substantial	13 (45%)
Religious affiliation		
	Christian	18 (62%)
	Muslim	2 (7%)
	Religious/spiritual, affiliation not clear	7 (24%)
	Non-religious	2 (7%)

**Table 2:** Summary of findings on aides’ experiences of job-based support.

Source of Job-Based Support	Summary of Findings		Sample Quotes
Coordinators	Aides identified the agency coordinators as occasional sources of support. However, participants emphasized both the value and inconsistency of coordinators acknowledging client death and the gaps in agencies’ provision of emotional support. Coordinators are sometimes seen as a good source of support if aides can quickly return to work after client loss. Some aides noted that coordinators could also help them to navigate time-off after client death, though they rarely did in practice.		“Sometimes the coordinator calls and she gives you support or she gives you sympathy because your patient died. Some of them don’t” (Participant 103, 17 years). “If I could receive a phone call from the coordinator on the case, a particular person within that unit, to keep in touch with me, “How are you feeling?” For some type of outreach to be made to me would help. I would feel less alone. Outreach from the agency that I’m working with, someone within the agency I’m working with. I would feel very much less alone in getting a phone call, you know, a text, a phone call to be, to make acknowledgement, you know, of where I am- communication, I would say, from someone within the agency.” (Participant 217, 2 years)
Agency Training	Aides viewed training as an important source of job-based support for navigating client death, though most existing training was described as insufficient. Some hospice aides described the value of lengthier hospice training.		“You need somebody that goes more in-depth with the classes and what you’re going through. It’s so much understanding, so much compassion [that’s needed].” (Participant 213, 8 years)
Agency Programs & Policies	Programmatic and policy actions that agencies might take related to client death were less prominent in aides’ descriptions of support, likely because these sources of support are less available, as research with agencies has demonstrated. Aides did mention a limited number of programmatic or policy supports that they wish agencies would provide. For instance, agency-based support groups (either in-person or online) for aides experiencing client death were mentioned. Another option described was a counselor, therapist or someone neutral, to meet with aides individually. As noted above, some aides also explicitly mentioned the lack of availability of paid time off policies following a client death and wished that such benefits were available to aides.		“I would think that they should get those aides [whose patients have died] and have debriefing with us. Go through it with us. And let us talk it out, because half of the time when patients die, we just move on. And so, we don’t need to move on. We need to talk. We need to share how we feel about it. […] Something is lingering in your heart. You need to talk. You understand? You need share, you need to express, and I believe that you need to—all of the aides should have groups like that. Bring us together and let us express, you know?” (Participant 216, 8 years) “I would like to the agency say if we need attention about that, but I don’t know if the agency don’t care about that or they don’t have, you know, mental professionals.” (Participant 003, 2 years) “They should have more support and they should give people the time—when the [patient dies]. Let them take the time off before you give another case like that. (Participant 204, 10 years)
Union	Aides spoke about two union sources of support: A bereavement phone line offered by the union and the union grievance process (for support with problems on the job in general). While promising, the bereavement phone line for aides who had lost a client was mentioned only by a single aide who had just learned about it in a hospice course. Notably, even when we asked about the union proactively, aides typically said that they did not know what support was offered, only acknowledging that the union perhaps could be a resource.		Regarding the phone line: “You can call them and they will give you that support and help you through the process.” (Participant 220, 5 years)

## Data Availability

Data are qualitative in nature and are detailed and contextualized. Making the data available thus risks making the participants identifiable.

## Supplementary Materials

The following supporting information can be downloaded at: https://www.mdpi.com/article/10.3390/ijerph19010367/s1, Supplemental File S1: In-Depth Interview Guides.
