# Supplementary material for: Expanding the Conceptualization of Support in Low-Wage Carework: The Case of Home Care Aides and Client Death
Source: Int J Environ Res Public Health. 2021 Dec 30;19(1):367. doi: 10.3390/ijerph19010367 (PMC8744702; doi:10.3390/ijerph19010367)
Supplement: Supplementary file 1 [file ijerph-19-00367-s001.zip › ijerph-1515795-supplementary.pdf]

Supplemental File 1: In-Depth Interview Guides

Section 1: Experiences with Patient Death & Support

1. Please start by telling me about how you began working in home care.

   Sub-questions:
   - How long have you been working in home care?

2. Now we'll move into talking about the patient deaths you've experienced. To start, can you tell me how many patient deaths you have experienced in your time working in home care?

3. Please think back to either the most recent experience you had with a patient dying or the most significant experience you've had with a patient dying. Tell me about this experience.

   Sub-questions:
   - What was your relationship with this client like?
   - How did he or she die?
   - How did the death impact you emotionally?
   - How did the death impact you in terms of hours, benefits, and financially?
   - How did the death impact your feelings about working as a home health aide?
   - Any other impacts that we have not discussed?
   - How long ago did your client die?

4. Next I'd like to learn more about the period following your patient's death.

   Sub-questions:
   - How much time did you take off from work, if any? What kinds of things did you do during this period of time (work or otherwise)?
   - When did you get your next case? What was that case like?
   - How did your approach to providing care change, if at all?

5. Next I'd like to learn more about the kinds of support that helped you through the period following this client's death. What kinds of support helped you?

   Sub-questions:
   - What kinds of emotional support did you receive? From whom? How did you seek this support? How did you receive this support (in person, phone, text, other)?
   - What kinds of financial support did you receive? From whom? How did you seek this support?
   - What other kinds of support did you receive? From whom? How did you seek this support?
   - For how long did you need these kinds of support?
   - What were the effects in your life of these forms of support?
   - What kinds of support did you need but not receive, if any?

6. When you think about your next client who may pass away, what kinds of support would most help you manage the stress of client death?

   Sub-questions:
   - Who will you reach out to? (Anyone who was not mentioned above?)
   - What kinds of support do you think you might seek? (Anything beyond what was mentioned above?)
   - What could help you feel more prepared?
   - What additional support could your agency or your union provide, if any?

Section 2: Reflections

7. In this next section, we'll be asking you to reflect on your experiences with death and dying. I'd like you to think about some of the things that are important at the end of life. This can be from your personal, religious, cultural, or professional experiences. What do you think is important at the end of life?

   Sub-questions:
   - In your opinion, what kinds of things are <u>helpful to or good</u> for a dying person, if anything? ("good" death)
   - In your opinion, what kinds of things are <u>problematic</u> for a dying person, if anything? ("bad" death)

8. I'm also curious about whether people who you know have similar ideas about the end of life. For instance, if you think about <u>your family members</u>, would they agree with these ideas about the end of life? (If so, why? If not, why not?)

   Sub-question:
   - If you think about <u>your friends</u>, would they agree with these ideas? (If so, why? If not, why not?)

9. This may or may not be related, but how would you describe your religious affiliation or spiritual approach, if any?

10. How important would you say religion and spirituality are to you?

    - Extremely important
    - Somewhat important
    - Not important

11. In what ways do you see religion and spirituality as being related to your thoughts about what's important at the end of life, if at all?

Questions Added during the Pandemic

1. Tell me about how you first realized that something was happening in terms of the pandemic.

2. Since that time, what are some of the changes that have taken place in your life?

3. How has work changed for you?

   Sub-questions:
   - How have your agency and/or union helped you while working during the pandemic? (Probe on experiences with PPE, training, support groups if this doesn't come up)
   - What have your interactions with coordinators or other staff from home care agencies been like?
   - How does it feel to be a home care worker during this time?
   - What has your experience working with any COVID-positive clients been like?

4. What kinds of support have you needed, wanted, and/or used during this time?

   Sub-questions (for any aide that cited religious affiliation in earlier interview):
   - What role has religion or spirituality played for you during this time?

5. What do you think others should know about the work that home care workers are doing during this period?

6. Have you experienced any client deaths since we spoke in the fall/winter? If yes, please tell me about one of those experiences.

   Sub-questions:
   - How long did you work with this client?
   - How did this client die?
   - What were the effects on you when this client died?
   - How have you managed since that client died? Was this different at all from how you've managed in the past?

7. As you know, one of the reasons we're doing this research is because we're curious about the kinds of support that agencies could offer that would be especially meaningful to aides in relation to client death, so I have two short questions for you on this topic:
   - If you learned that another home care agency had an especially well-trained staff of supervisors/coordinators who were all thoughtful, caring, and sensitive, would you consider working for that agency instead? (Probe on reasons)
   - If you learned that another home care agency was offering 2 days of paid leave specifically for the period of time after your client died, would you consider working for that agency instead? (Probe on reasons)

8. Is there anything else that we didn't talk about on what it's been like to work during the pandemic that would be helpful for me to know?
